# Supplementary material for: Clinical value of metagenomic next-generation sequencing in screening oropharyngeal colonization in patients undergoing allogeneic hematopoietic stem cell transplantation: a prospective observational study
Source: Microbiol Spectr. 2025 May 27;13(7):e00028-25. doi: 10.1128/spectrum.00028-25 (PMC12210989; doi:10.1128/spectrum.00028-25)
Supplement: Supplemental material — Fig. S1 and S2; Tables S1 to S6. [file spectrum.00028-25-s0001.docx]

# Supplementary materials


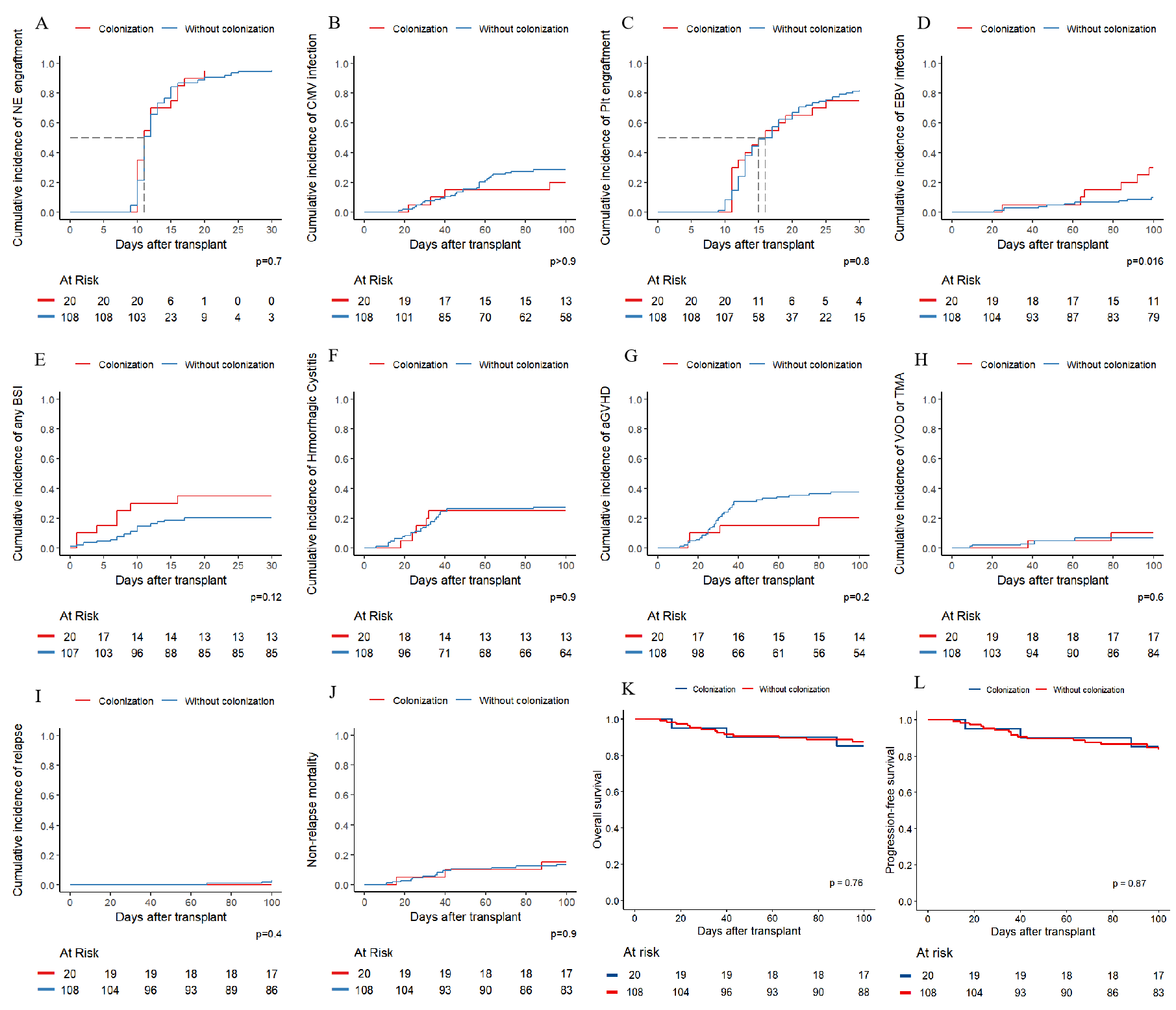


Supplementary Figure 1. Comparison of post-transplant complications and prognoses between colonization and non-colonization patients detected by traditional culture methods, including (A) neutrophil engraftment, (B) platelet engraftment, (C) CMV infection, (D) EBV infection, (E) BSI, (F) aGVHD, (G) hemorrhagic cystitis, (H) VOD or TMA, (I) relapse, (J) NRM, (K) OS, and (L) PFS.


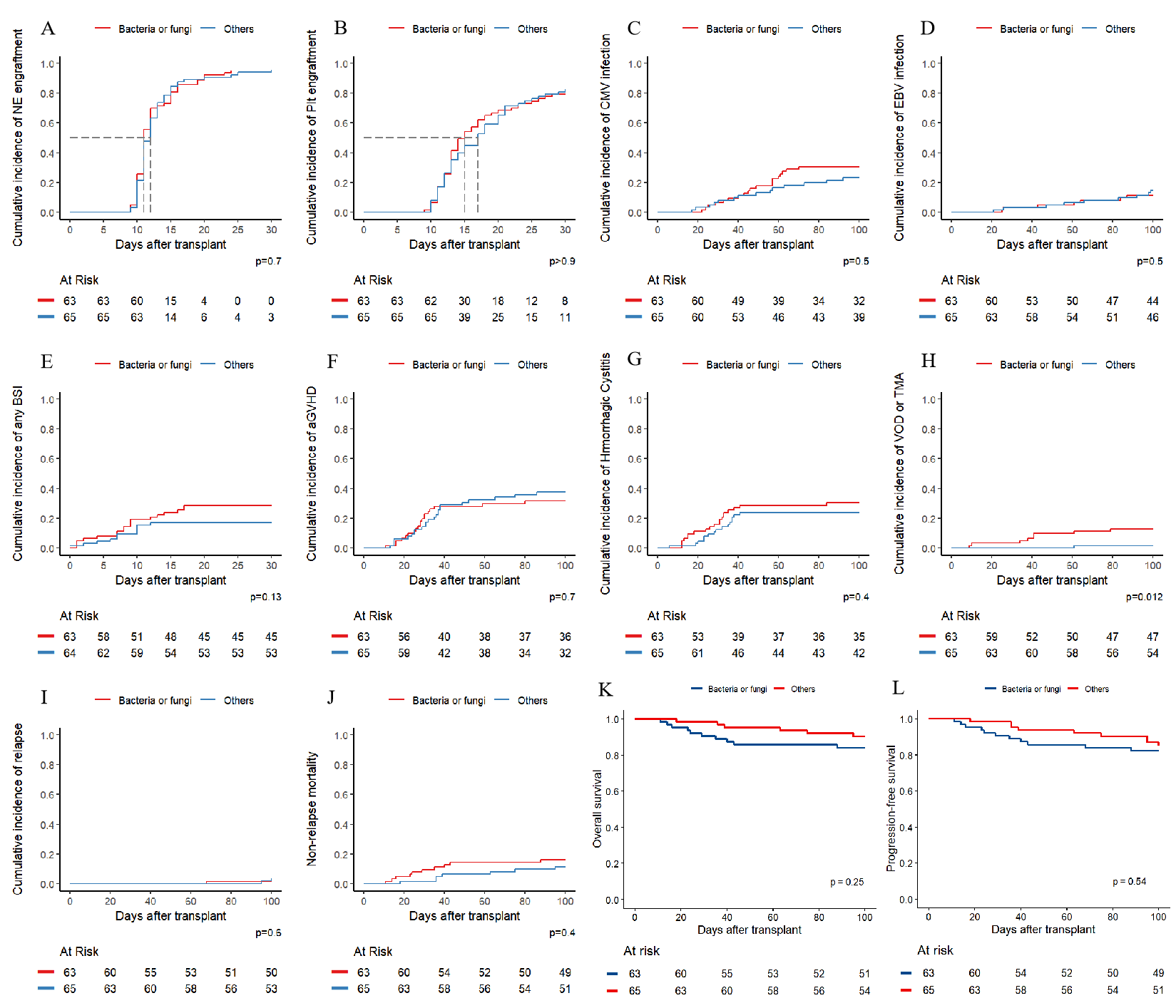


Supplementary Figure 2. Comparison of post-transplant complications and prognoses between patients with and without bacteria or fungi detected by a pre-transplant oropharyngeal mNGS test, including (A) neutrophil engraftment, (B) platelet engraftment, (C) CMV infection (D) EBV infection, (E) BSI, (F) aGVHD, (G) hemorrhagic cystitis, (H) VOD or TMA, (I) relapse, (J) NRM, (K) OS, and (L) PFS.

Supplementary Table 1. Overview of the detection of oropharyngeal Enterobacteriaceae colonization in 23 patients.

| No. | mNGS | Culture |
| --- | --- | --- |
| 4 | KP | / |
| 6 | *E. cloacae* | *E. cloacae* |
| 8 | KP | KP |
| 13 | *E. cloacae* | / |
| 22 | KP | KP |
| 32 | KP | / |
| 43 | KP | / |
| 53 | KP | / |
| 55 | KP | / |
| 57 | KP | / |
| 85 | *E. cloacae* | / |
| 89 | *E. cloacae* | / |
| 90 | KP, *E. cloacae* | / |
| 93 | KP | KP |
| 95 | *E. cloacae* | / |
| 98 | *E. cloacae* | KP |
| 103 | *E. cloacae* | / |
| 106 | *E. coli* | / |
| 107 | KP | / |
| 113 | KP | / |
| 119 | *E. cloacae* | *E. cloacae* |
| 126 | *E. coli* | / |
| 128 | KP | / |

KP，*Klebsiella pneumoniae*；*E. cloacae，Enterobacter cloacae*；*E. coli，Escherichia coli.*

Supplementary Table 2. Comparison of clinical characteristics between Enterobacteriaceae colonization and non-Enterobacteriaceae colonization groups.

|  | **Enterobacteriaceae**  **(*n* = 23)** | **Non-Enterobacteriaceae**  **(*n* = 105)** | ***p* value** |
| --- | --- | --- | --- |
| **Male gender (%)** | 13 (56.5) | 51 (48.6) | 0.645 |
| **Age (median [IQR])** | 53 (44,59) | 42 (29,51) | **0.002** |
| **Underlying diseases (%)** |  |  | **0.036** |
| AA/PNH | 4 (17.4) | 16 (15.2) |  |
| ALL | 2 (8.7) | 10 (9.5) |  |
| AML | 15 (65.2) | 36 (34.3) |  |
| CML | 0 (0.0) | 5 (4.8) |  |
| Lymphoma | 2 (8.7) | 3 (2.9) |  |
| MDS | 0 (0.0) | 26 (24.8) |  |
| MPAL | 0 (0.0) | 5 (4.8) |  |
| Others | 0 (0.0) | 4 (3.8) |  |
| **HCT-CI (median [IQR])** | 1 (1, 2) | 1 (1, 2) | 0.971 |
| **Days from diagnosis to transplantation (median [IQR])** | 181 (100, 400) | 166 (95, 329) | 0.973 |
| **Times of chemotherapy**  **(median [IQR])** | 3 (2, 7) | 3 (1, 4) | 0.256 |
| **HLA antibody positive (%)** | 8 (34.8) | 37 (35.2) | 1.000 |

Supplementary Table 3. Competitive risk model of pre-transplant colonization-associated EBV infection identified using a culture.

| Characteristics | Univariate Analysis | | |  | Multivariate Analysis | | |
| --- | --- | --- | --- | --- | --- | --- | --- |
|  | HR | CI95 | *p*-value |  | HR | CI95 | *p*-value |
| **Colonization identified by culture** | 3.12 | 1.20-8.11 | **0.020** |  | 4.38 | 1.46-13.20 | **0.009** |
| **Gender** |  |  |  |  |  |  |  |
| Male |  |  |  |  |  |  |  |
| Female | 0.31 | 0.12-0.83 | **0.019** |  | 0.91 | 0.31-2.69 | 0.860 |
| **Age** |  |  |  |  |  |  |  |
| ≤50 |  |  |  |  |  |  |  |
| >50 | 0.99 | 0.37-2.83 | 0.990 |  |  |  |  |
| **Lines of chemotherapy** |  |  |  |  |  |  |  |
| ≤3 |  |  |  |  |  |  |  |
| >3 | 0.81 | 0.31-2.11 | 0.670 |  |  |  |  |
| **Days from Diagnosis to HSCT** |  |  |  |  |  |  |  |
| ≤167 |  |  |  |  |  |  |  |
| >167 | 1.16 | 0.47-2.90 | 0.750 |  |  |  |  |
| **Diagnosis** |  |  |  |  |  |  |  |
| AA/PNH/AA-PNH |  |  |  |  |  |  |  |
| ALL | 0.00 | 0.00-0.00 | < 0.001 |  |  |  |  |
| AML | 0.23 | 0.07-0.77 | **0.017** |  | 0.17 | 0.03-0.90 | **0.036** |
| Lymphoma | 0.66 | 0.09-5.04 | 0.690 |  |  |  |  |
| MDS | 0.32 | 0.08-1.25 | 0.100 |  |  |  |  |
| MPAL | 0.00 | 0.00-0.00 | < 0.001 |  |  |  |  |
| Others | 1.10 | 0.27-4.48 | 0.890 |  |  |  |  |
| **HCT-CI** | 0.52 | 0.28-0.97 | **0.041** |  | 0.47 | 0.22-0.97 | **0.041** |
| **HLA antibody positive** | 0.10 | 0.01-0.60 | **0.012** |  | 0.04 | 0.00-0.39 | **0.006** |
| **ATG use** | 1.82 | 0.23-14.1 | 0.570 |  |  |  |  |
| **Donor** |  |  |  |  |  |  |  |
| Haploid |  |  |  |  |  |  |  |
| MMUD | 0.83 | 0.27-2.56 | 0.950 |  |  |  |  |
| MSD | 0.92 | 0.12-7.08 | 0.940 |  |  |  |  |
| MUD | 0.86 | 0.12-6.00 | 0.880 |  |  |  |  |
| **ABO compatibility** | 0.65 | 0.26-1.62 | 0.350 |  |  |  |  |
| **Graft source** |  |  |  |  |  |  |  |
| BM |  |  |  |  |  |  |  |
| PB+BM | 6.41 | 0.46-88.3 | 0.170 |  |  |  |  |
| UCB | 0.00 | 0.00-0.00 | < 0.001 |  |  |  |  |
| **Cord blood use** | 0.29 | 0.12-0.69 | **0.005** |  | 0.49 | 0.20-1.21 | 0.120 |
| **Total MNC (10E8/kg)** | 1.06 | 1.01-1.12 | **0.019** |  | 1.02 | 0.96-1.08 | 0.490 |
| **Total CD34+ (10E6/kg)** | 1.00 | 0.91-1.10 | 0.960 |  |  |  |  |
| **Total CD3+ (10E6/kg)** | 0.85 | 0.52-1.38 | 0.500 |  |  |  |  |

Supplementary Table 4. Competitive risk model of pre-transplant oropharyngeal colonization-associated VOD or TMA identified using mNGS.

| Characteristics | Univariate Analysis | | |  | Multivariate Analysis | | |
| --- | --- | --- | --- | --- | --- | --- | --- |
|  | HR | CI95 | *p*-value |  | HR | CI95 | *p*-value |
| **Colonization identified by mNGS** | 3.81 | 1.52-9.58 | **0.004** |  | 3.84 | 1.49-9.89 | **0.005** |
| **Gender** |  |  |  |  |  |  |  |
| Male |  |  |  |  |  |  |  |
| Female | 3.72 | 1.14-12.1 | **0.029** |  | 3.45 | 1.06-11.2 | **0.040** |
| **Age** |  |  |  |  |  |  |  |
| ≤ 50 |  |  |  |  |  |  |  |
| > 50 | 2.55 | 0.83-7.83 | 0.010 |  |  |  |  |
| **Lines of chemotherapy** |  |  |  |  |  |  |  |
| ≤ 3 |  |  |  |  |  |  |  |
| > 3 | 1.68 | 0.63-4.50 | 0.300 |  |  |  |  |
| **Days from Diagnosis to HSCT** |  |  |  |  |  |  |  |
| ≤ 167 |  |  |  |  |  |  |  |
| > 167 | 1.20 | 0.43-3.32 | 0.720 |  |  |  |  |
| **Diagnosis** |  |  |  |  |  |  |  |
| ALL |  |  |  |  |  |  |  |
| AA/PNH/AA-PNH | 0.00 | 0.00-0.00 | < 0.001 |  |  |  |  |
| AML | 0.59 | 0.12-2.84 | 0.510 |  |  |  |  |
| Lymphoma | 2.00 | 0.20-19.7 | 0.550 |  |  |  |  |
| MDS | 0.37 | 0.06-2.12 | 0.260 |  |  |  |  |
| MPAL | 1.46 | 0.12-17.8 | 0.770 |  |  |  |  |
| Others | 0.00 | 0.00-0.00 | < 0.001 |  |  |  |  |
| **HCT-CI** | 0.90 | 0.57-1.42 | 0.650 |  |  |  |  |
| **HLA antibody positive** | 1.21 | 0.36-4.14 | 0.760 |  |  |  |  |
| **ATG use** | 0.96 | 0.13-7.06 | 0.970 |  |  |  |  |
| **Donor** |  |  |  |  |  |  |  |
| Haploid |  |  |  |  |  |  |  |
| MMUD | 0.50 | 0.14-1.86 | 0.300 |  |  |  |  |
| MSD | 0.00 | 0.00-0.00 | < 0.001 |  |  |  |  |
| MUD | 2.71 | 0.69-10.60 | 0.150 |  |  |  |  |
| **ABO compatibility** | 1.04 | 0.38-2.83 | 0.940 |  |  |  |  |
| **Graft source** |  |  |  |  |  |  |  |
| BM |  |  |  |  |  |  |  |
| PB+BM | 0.00 | 0.00-0.00 | < 0.001 |  |  |  |  |
| UCB | 1.83 | 0.53-6.34 | 0.340 |  |  |  |  |
| **Cord blood use** | 2.08 | 1.10-3.91 | **0.023** |  | 1.96 | 0.97-3.96 | 0.059 |
| **Total MNC (10E8/kg)** | 1.01 | 0.94-1.10 | 0.720 |  |  |  |  |
| **Total CD34+ (10E6/kg)** | 0.97 | 0.90-1.05 | 0.430 |  |  |  |  |
| **Total CD3+ (10E6/kg)** | 1.41 | 0.70-2.81 | 0.340 |  |  |  |  |

Supplementary Table 5. Competitive risk model of pre-transplant oropharyngeal Enterobacteriaceae colonization-associated VOD or TMA identified using mNGS.

| Characteristics | Univariate Analysis | | |  | Multivariate Analysis | | |
| --- | --- | --- | --- | --- | --- | --- | --- |
|  | HR | CI95 | *p*-value |  | HR | CI95 | *p*-value |
| **Enterobacteriaceae colonization identified by mNGS** | 2.78 | 0.89-8.61 | **0.077** |  | 2.66 | 0.85-8.31 | 0.092 |
| **Gender** |  |  |  |  |  |  |  |
| Male |  |  |  |  |  |  |  |
| Female | 3.72 | 1.14-12.1 | **0.029** |  | 3.38 | 1.09-10.5 | **0.035** |
| **Age** |  |  |  |  |  |  |  |
| ≤ 50 |  |  |  |  |  |  |  |
| > 50 | 2.55 | 0.83-7.83 | 0.010 |  |  |  |  |
| **Lines of chemotherapy** |  |  |  |  |  |  |  |
| ≤ 3 |  |  |  |  |  |  |  |
| > 3 | 1.68 | 0.63-4.50 | 0.300 |  |  |  |  |
| **Days from Diagnosis to HSCT** |  |  |  |  |  |  |  |
| ≤ 167 |  |  |  |  |  |  |  |
| > 167 | 1.20 | 0.43-3.32 | 0.720 |  |  |  |  |
| **Diagnosis** |  |  |  |  |  |  |  |
| ALL |  |  |  |  |  |  |  |
| AA/PNH/AA-PNH | 0.00 | 0.00-0.00 | < 0.001 |  |  |  |  |
| AML | 0.59 | 0.12-2.84 | 0.510 |  |  |  |  |
| Lymphoma | 2.00 | 0.20-19.7 | 0.550 |  |  |  |  |
| MDS | 0.37 | 0.06-2.12 | 0.260 |  |  |  |  |
| MPAL | 1.46 | 0.12-17.8 | 0.770 |  |  |  |  |
| Others | 0.00 | 0.00-0.00 | < 0.001 |  |  |  |  |
| **HCT-CI** | 0.90 | 0.57-1.42 | 0.650 |  |  |  |  |
| **HLA antibody positive** | 1.21 | 0.36-4.14 | 0.760 |  |  |  |  |
| **ATG use** | 0.96 | 0.13-7.06 | 0.970 |  |  |  |  |
| **Donor** |  |  |  |  |  |  |  |
| Haploid |  |  |  |  |  |  |  |
| MMUD | 0.50 | 0.14-1.86 | 0.300 |  |  |  |  |
| MSD | 0.00 | 0.00-0.00 | < 0.001 |  |  |  |  |
| MUD | 2.71 | 0.69-10.60 | 0.150 |  |  |  |  |
| **ABO compatibility** | 1.04 | 0.38-2.83 | 0.940 |  |  |  |  |
| **Graft source** |  |  |  |  |  |  |  |
| BM |  |  |  |  |  |  |  |
| PB+BM | 0.00 | 0.00-0.00 | < 0.001 |  |  |  |  |
| UCB | 1.83 | 0.53-6.34 | 0.340 |  |  |  |  |
| **Cord blood use** | 2.08 | 1.10-3.91 | **0.023** |  | 1.83 | 1.00-3.35 | 0.050 |
| **Total MNC (10E8/kg)** | 1.01 | 0.94-1.10 | 0.720 |  |  |  |  |
| **Total CD34+ (10E6/kg)** | 0.97 | 0.90-1.05 | 0.430 |  |  |  |  |
| **Total CD3+ (10E6/kg)** | 1.41 | 0.70-2.81 | 0.340 |  |  |  |  |

Supplementary Table 6. Detection of resistance genes in CRE and prognoses of patients with oropharyngeal CRE colonization.

| No. | Isolate | Beta-Lactams’ resistance genes | Aminoglycosides resistance genes | Quinolone resistance genes | Colistin  resistance genes | BSI (isolate) | Outcome,  causes of death |
| --- | --- | --- | --- | --- | --- | --- | --- |
| 1 | *E. cloacae* | NDM-1 | AAC (3)-IIe | QnrB1 | MCR | No | Alive |
| 2 | *E. cloacae* | NDM-1  SHV-12 | / | / | / | Yes (*E. cloacae*) | Death 25 days after HSCT,  Severe infection and TMA |
| 3 | *E. cloacae* | NDM-1 | AAC (3)-IId | / | / | Yes (*E. cloacae*) | Alive |
| 4 | KP^*^ | / | / | / | / | Yes (KP) | Death 16 days after UCBT,  Severe infection |
| 5 | KP* | / | / | / | / | Yes (KP) | Death 88 days after UCBT,  Severe infection and TMA |

^*^Two cases of KP were identified as CRKP via culture and drug sensitivities.
